# The relationship between (stigmatizing) views and lay public preferences regarding tuberculosis treatment in the Eastern Cape, South Africa

**DOI:** 10.1186/1475-9276-10-2

**Published:** 2011-01-14

**Authors:** Jane M Cramm, Anna P Nieboer

**Affiliations:** 1 Institute of Health Policy and Management, Erasmus University Rotterdam, P.O. Box 1738, 3000 DR Rotterdam, the Netherlands

## Abstract

**Background:**

Tuberculosis (TB) and human immune virus/acquired immune deficiency syndrome (HIV/AIDS) stigmas affect public attitudes toward TB treatment and policy. This study examined 'stigmatizing' ideas and the view that 'TB patients should line-up in the chronic illness queue' in relation to preferences and attitudes toward TB treatment.

**Methods:**

Data were gathered through a survey administered to respondents from 1,020 households in Grahamstown. The survey measured stigmatization surrounding TB and HIV/AIDS, and determined perceptions of respondents whether TB patients should queue with other chronically ill patients. Respondents selected support and treatment options they felt would benefit TB patients. Statistical analysis identified the prevalence of TB and HIV/AIDS stigmas. Logistic regression analyses explored associations between stigmatizing ideas, views regarding TB patients in the chronic illness queue, and attitudes toward support and treatment.

**Results:**

Respondents with TB stigmatizing ideas held positive attitudes toward volunteer support, special TB queues, and treatment at clinics; they held negative attitudes toward temporary disability grants, provision of information at work or school, and treatment at the TB hospital. Respondents who felt it beneficial for TB patients to queue with other chronically ill patients conversely held positive attitudes toward provision of porridge and disability grants, and treatment at the TB hospital; they held negative attitudes toward volunteer support, special TB queues, information provision at work or school, and treatment at clinics.

**Conclusion:**

These results showed that two varying views related to visibility factors that expose patients to stigmatization (one characterized by TB stigma, the other by the view that TB patients should queue with other chronically ill patients) are associated with opposing attitudes and preferences towards TB treatment. These opposing attitudes complicate treatment outcomes, and suggest that complex behaviors must be taken into account when designing health policy.

## Background

Tuberculosis (TB) is a major cause of illness and death worldwide, particularly in low- and middle-income countries where it is fueled by human immune virus/acquired immune deficiency syndrome (HIV/AIDS). TB is the most common secondary infection for the approximately 5.5 million South Africans living with HIV/AIDS. About 700 per 100,000 of the national population are infected with TB, classifying it as a serious epidemic [1]. It is particularly endemic in the Eastern Cape, a South African province characterized by high levels of poverty and unemployment. Its cure rate of 41% is well below that recommended by the World Health Organization (85%) [1,2]. WHO's 2006 response to 9.2 million new cases and 1.7 million TB-related deaths worldwide was to launch a "Stop TB" strategy [1] that built on the Directly Observed Treatment Short Course (DOTS) launched in 1995. More than 22 million patients have been treated under DOTS-based services [3] but the results seem to lag behind their aim [1,2,4].

Disease-related stigmas negatively influence treatment, including that of TB [5-7]. TB stigmatization is interesting in that the initial social shunning was largely eliminated with a TB cure, but stigmas re-emerged when it became a marker for HIV/AIDS [7-9]. The effect was amplified when the cost effective combination of TB and HIV/AIDS treatment was chosen as a recommended strategy [3,9]. Disease-related stigmas also negatively affect public attitudes toward prevention, service provision, and health-related policies. A study on stigmas has indicated that people with less stigmatizing ideas were significantly more favorably responsive to disease-related governmental policies [10]. Availability of treatment, however, does not necessarily translate to seeking or adhering to it. The Botswanan government, for example, recently began offering a free, widely publicized, and easily accessible antiretroviral (ARV) treatment to all citizens with AIDS but only 15% of eligible citizens took part in the program [11]. Different views related to visibility factors that expose patients to stigmatization may lead to varying attitudes and preferences towards TB treatment. In a qualitative study on TB stigma in South Africa Møller & Erstad (2007) found that visibility factors around TB treatment provision are related to stigma. In South African townships there are often long waiting queues at the clinics and TB patients mention having problems with presenting at the TB clinics for treatment because they want to avoid being seen in a TB queue. In addition, their study findings indicate that hospitalization in the local TB hospital, and arrangements in health clinics that group HIV/AIDS and TB together, have become markers of stigma. As a result to avoid being stigmatized, people reportedly avoided any behavior that might identify them as being infected with TB or AIDS. Persons with TB symptoms were tempted to 'hide' their status, for example, people avoided joining queues and entering rooms reserved for AIDS patients in clinics and people were reluctant to be admitted to the local TB hospital [7]. Thus, TB- and HIV/AIDS-associated stigmas influence both attitudes and behavior toward treatment. The persistence of HIV- and TB-related stigmatization - antiretroviral medications and TB treatment programs notwithstanding - reminds us that efforts to reduce it must be renewed. An intervention that recasts TB and HIV as chronic manageable conditions [12] may be one such effort.

Although research indicates that public attitudes and health care policy responses are strongly influenced by perceptions of illness [6] and that others' attitudes influence how individuals define and act upon symptoms and life crises [13], it remains unclear how disease-related stigmas affect treatment access and compliance. Since the Eastern Cape has a high incidence/prevalence of TB and one of the worst cure rates in South Africa, its inhabitants can be considered experienced in relating to TB. In this study, we thus seek to identify the pervasiveness of TB and HIV/AIDS stigmas, as well as attitudes toward treatment. We also investigate the relationship between two views, one characterized by stigmatization and the other (more non-stigmatizing view) that TB patients should queue with other chronically ill patients. Understanding of the relationship between (stigmatizing) views and lay public TB treatment preferences may be helpful to TB program design and better treatment outcomes.

## Methods

The Eastern Cape is a South African province characterized by high levels of poverty, unemployment and where TB is endemic [7]. We used data from a November, 2007 survey of 1,020 Grahamstown East/Rhini households. The median age of respondents was 38 and almost three quarters (73%) were women. Just over half were single (52%), a third married (33%) and the others widowed (9%) or separated/divorced (6%). Forty percent had completed some secondary education and 18% had matriculated. Approximately 7% had received post-matriculation education and training. Only 8% had no formal schooling.

TB prevalence was assessed by a dichotomised lifetime tuberculosis item that stated, 'There has been a case of TB in this household'. We did not assess individual-level experience with TB because the interview was held face-to-face; given the stigmatisation of TB, we felt that respondents might not answer such questions truthfully. About one third (32.3%) of respondents claimed to (had) have a TB case in the household in accordance with TB prevalence statistics [3].

### Ethical approval

This study was approved by the Rhodes University Ethical Standards Committee. All personal identifiers have been removed or disguised such that the person(s) are not identifiable.

### Subjects and sampling design

An area-stratified sampling design was applied. Households in the 23 neighborhoods of Rhini, a suburb of Grahamstown, were randomly selected in proportion to the total number of households in each neighborhood. Moving systematically through each neighborhood from a random starting point, every tenth household was included in the sample. All households in all areas of Rhini thus had an equal probability of survey inclusion.

Eligible respondents were over 17 years old and had resided in Rhini for at least 6 months in the past year. One respondent per household was chosen using a Kish grid to ensure that all eligible persons in the household had an equal probability of survey inclusion. Identified respondents were interviewed immediately if they consented or arrangements were made to conduct the interview later. Up to four visits to the house were made to interview respondents who were initially absent. Interviews were obtained in 1,020 of the 1,042 households (97.9%). The 22 respondents dropped from the sample either (1) could not be contacted by the fourth visit to the house, or were (2) too old, (3) too ill, or (4) unwilling.

Trained Development Research Africa and local interviewers conducted the interviews. Nearly all questions were closed-ended items for which a set of response options was supplied. A detailed description of the study population can be found in the Institute of Social and Economic Research, Research Report Series No. 14 & No. 15 [14,15].

### Instruments

We have good reason to believe that the concepts underlying the previously developed AIDS-related stigma scale (e.g., stained social identity, blame, shamefulness, avoidance, social sanction) apply to stigmatization of TB patients as well. The brief nine-item scale has been tested in several South African communities, is internally consistent and reliable, and has a Cronbach's alpha (α) of 0.75 [16]. One item exhibiting a low correlation with the total score that reduced the internal consistency of the scale was discarded. The resulting eight-item AIDS-related stigma scale was used to assess the stigmatizing ideas of respondents regarding HIV (Cronbach's α of 0.77) and TB (Cronbach's α of 0.74). Respondents could either agree or disagree with each item (appendix). We calculated TB stigma and HIV stigma using count scores of correct answers (range 0-8). We also added a statement concerning visibility: Would TB patients stay on treatment if they could queue together with chronic disease patients?

In the Eastern Cape, TB treatment can be obtained at a TB hospital, a clinic, or at home. In the case of home care, either a family member or a DOTS volunteer provides medicine daily. Visibility, or TB identifiability, is an important factor with respect to stigmatization; the survey thus asked respondents to state preferences for treatment location and medicine delivery mode.

Most surveys of lay public attitudes use a rating approach [17] in which all items are equally important and therefore lack the prioritizing that would facilitate treatment decisions [18]. Here, we opted for the more realistic priority setting with a ranking approach [17,19].

A 2006 focus group identified five TB treatment support types available in the Rhini area [7]. TB patients can be paired with a DOTS volunteer for support; food assistance in the form of porridge can be provided, which ensures that medication is not taken on an empty stomach; special queues or rooms are made available at some clinics to reduce waiting time for TB patients who collect medication themselves; TB patients are provided a temporary disability grant, ensuring financial independence while on treatment; and volunteers will inform TB patients' employers or schools that the disease is not infectious when it is being treated. Respondents were asked to select up to two support types felt to be the most helpful to patients and their families.

### Data Analysis

The analysis consisted of three parts. To determine the level of stigmatizing ideas and test the view that TB treatment should be integrated with chronic illnesses we first used a descriptive analysis to characterize the prevalence of the views. Second, we performed a logistic regression analysis to test whether stigmatizing ideas and the view of queuing TB patients together with chronically ill patients are associated with different attitudes and preferences toward TB treatment and support types (it does not help to put TB patients in a queue with other chronically ill patients = 0; it does help to put TB patients in a queue with other chronically ill patients = 1). We dichotomized stigma for use in the logistic regression analysis into scores of 0 versus scores of > 0 (not stigmatizing ideas = 0; stigmatizing ideas = 1). Because stigma was an index based on the number of correct answers, we dichotomized it to conduct logistic regression analyses rather than multivariate analyses and scale scores. For regression analyses, we reported odd ratio (OR) values. Last, correlation and logistic regression analyses were performed to test whether differences in TB experience, age, gender, marital status and education are associated with different attitudes and preferences toward TB treatment and treatment assistance types. Significant variables from the correlation analyses were entered into the logistic regression model.

## Results

The prevalence of stigmatizing ideas regarding HIV/AIDS in this community was higher (mean 0.93) than that of stigmatizing ideas surrounding TB (mean 0.52). Pearson correlations for stigmatizing ideas of TB and HIV/AIDS revealed a relation (*r *= 0.66; *p *≤ 0.001). Of the 1,020 respondents, 70% believed it would be beneficial to have TB patients queuing with chronic diseases. When we look at the respondents who agree or disagree with the belief that it would be beneficial to have TB patients queuing with chronic diseases, we found that they differ on the TB stigma scale. Respondents who felt it would be beneficial to have TB patients queuing with chronic diseases held less stigmatizing views toward TB patients. They did not differ on HIV stigma.

Logistic regression analyses examined whether stigmatizing ideas and/or the view that TB patients should be queuing with other chronically ill patients are associated with different attitudes toward support and treatment preferences. No significant relations were found between HIV/AIDS stigmas and attitudes toward support and treatment preferences. TB stigmatizing ideas were significantly associated with a negative attitude toward providing TB patients disability grants and allaying fears of contagion at work or school; they were significantly related to a positive attitude toward DOTS volunteers and special queues for TB patients at the clinics. The view that TB patients would benefit from queuing with other chronically ill patients was associated with a positive attitude toward food assistance and disability grants. It revealed a negative attitude toward support from DOTS volunteers and providing information about non-contagiousness to employer or school (table 1).

**Table 1:** Logistic regression analyses of TB treatment assistance preferences and TB stigmatizing ideas, HIV/AIDS stigmatizing ideas and the view that TB patients should be queued with other chronically ill patients.

	Assigning a DOTS volunteer to support them while on treatment	Providing porridge so TB patients do not take their medicine on an empty stomach	Assigning special queues or a special room at clinics	Giving TB patients a temporary disability grant so they can be financially independent while on treatment	Contacting people at work or school to inform them that the patient is not infectious because he/she is on treatment
	
	Adjusted OR	Adjusted OR	Adjusted OR	Adjusted OR	Adjusted OR
TB stigma	**1.230 (1.084-1.449)**	1.075 (0.933-1.225)	**1.169 (1.000-1.354)**	**0.708 (0.600-0.826)**	**0.852 (0.671-1.009)**
HIV/AIDS Stigma	1.122 (0.992-1.316)	0.975 (0.828-1.104)	0.960 (0.838-1.120)	0.982 (0.831-1.109)	0.937 (0.786-1.132)
TB patients queued with chronically ill	**0.756 (0.583-0.974)**	**1.442 (1.094-1.879)**	0.873 (0.660-1.177)	**1.667 (1.283-2.083)**	**0.710 (0.517-0.971)**
Constant	0.950	1.161	**0.364**	**0.508**	**0.415**
					
Model χ^2^	**x^2 ^= 22.443**	**x^2 ^= 8.062**	x^2 ^= 5.295	**x^2 ^= 42.181**	**x^2 ^= 8.475**
-2 log likelihood	1381,757	1308.976	1126.231	1361.622	984.000
Nagelkerke R^2^	0.029	0.011	0.008	0.054	0.013

Notes: Figures in bold are statistically significant at p < 0.05.

Not stigmatizing ideas = 0; stigmatizing ideas = 1.

It does not help to put TB patients in a queue with other chronically ill patients = 0; it does help to put TB patients in a queue with other chronically ill patients = 1.

The tables stating the logistic regression models also adjust for gender and education.

Lay public attitudes indicated that TB stigmatizing ideas are related to a positive attitude toward treatment at clinics and a negative attitude toward treatment at the TB hospital. The view that TB patients should be queuing with the chronically ill predicted the opposite: a positive attitude toward TB treatment at the hospital and a negative attitude toward treatment at the clinics (table 2).

**Table 2:** Logistic regression analyses of TB treatment preferences and TB stigmatizing ideas, HIV/AIDS stigmatizing ideas and the view that TB patients should be queued up with other chronically ill patients.

	TB hospital	Clinic	Family member collecting medicine	DOTS volunteer collecting medicine
	
	Adjusted OR	Adjusted OR	Adjusted OR	Adjusted OR
TB stigma	**0.784 (0.657-0.899)**	**1.326 (1.153-1.537)**	1.030 (0.838-1.294)	0.841 (0.684-1.056)
HIV/AIDS Stigma	1.122 (0.957-1.276)	0.975 (0.836-1.121)	0.966 (0.778-1.214)	0.859 (0.712-1.058)
TB patients queued with chronically ill	**1.135 (0.997-1.267)**	**0.950 (0.825-1.082)**	0.772 (0.522-1.130)	0.869 (0.630-1.210)
Constant	**0.339**	**0.643**	**0.167**	**0.289**
Model χ^2^	**x^2 ^= 26.003**	**x^2 ^= 21.687**	x^2 ^= 1.817	**x^2 ^= 7.646**
-2 log likelihood	1340.191	1275.412	687.333	901.778
Nagelkerke R^2^	0.034	0.029	0.004	0.013

Notes: Figures in bold are statistically significant at p < 0.05.

Not stigmatizing ideas = 0; stigmatizing ideas = 1.

It does not help to put TB patients in a queue with other chronically ill patients = 0; it does help to put TB patients in a queue with other chronically ill patients

The tables stating the logistic regression models also adjust for gender and education.

Correlation and logistic regression analyses were performed to test for different attitudes and preferences toward TB treatment and treatment assistance types in households with and without TB experience. About one third (32.3%) of respondents claimed to have (had) a TB case in the household. Correlation analyses revealed that only gender and education are associated with TB treatment and treatment assistance types. We found no significant differences in outcomes with age, marital status and households with and without TB experience. When we entered gender and education into the logistic regression models we only found an association between education and contacting people at work or school to inform them that the patient is not infectious because he/she is on treatment. Higher educated held a more positive attitude toward this type of assistance. Data are not presented but information is available on request.

## Discussion

We investigated lay perceptions on TB treatment in the Eastern Cape and tried to identify the pervasiveness of stigmas and the relationships between (stigmatizing) ideas and TB treatment attitudes and preferences. Because this area has a high incidence/prevalence of TB and one of the worst cure rates of South Africa [1,2], its inhabitants can be considered experts when it comes to matters concerning TB. Our study results are thus relevant to possible improvement of treatment outcomes. The most salient aspect of the study is that two views (one characterized by TB stigma, the other by the view that TB patients should queue with other chronically ill patients) are associated with opposite attitudes and preferences towards TB treatment.

### Opposing attitudes and preferences toward TB treatment

In this study HIV stigmatizing ideas showed no significant attitudes in relation with attitudes and preferences toward TB treatment in addition to TB stigmatizing ideas, probably because the correlation analysis revealed that the stigmas are related to each other. Other research has also shown how visible signs of TB trigger HIV stigmatization and even claim that a new disease stigma has emerged, namely the TB-HIV stigma [8]. TB stigmatizing ideas were associated with positive attitudes toward treatment at the clinic and negative attitudes toward treatment at the hospital, the opposite was associated with the preference to queue TB patients with other chronically ill individuals.

Theoretically, all TB patients are assigned a treatment supervisor. His or her role at the hospital and clinics is minimal, however, compared to that of family members or volunteers who distribute medicine. TB patients who personally collect their medication at a hospital or clinic may be identified as TB- or HIV/AIDS-infected (and therefore stigmatized), especially when TB and HIV/AIDS treatments are combined. Home visits from a DOTS volunteer also increases neighborhood awareness of the presence of TB in the household and consequently may lead to stigmatizing, which, in turn, has repercussion on TB treatment. Our results showed that respondents with TB stigmatizing ideas or the view that TB patients should queue with other chronically ill patients indeed are associated with different attitudes toward TB treatment. There seems to be no single solution. Combined TB and HIV/AIDS treatment presents another problem. Some views of the lay public (e.g., views regarding combined or separate TB queues) might lead to negative attitudes toward combined TB and HIV/AIDS treatment, resulting in a disincentive to seek treatment [20]. Such varying attitudes could explain the Botswanan example, where only 15% of citizens with AIDS participated in the free ARV treatment program [11].

### Opposing attitudes and preferences toward TB treatment assistance

For other assistance types, the two views relate to contrasting attitudes. While TB stigmatizing ideas are related to a positive attitude toward assigning DOTS volunteers, the view that TB patients should queue with the chronically ill is associated with a negative attitude toward DOTS volunteers. The view that TB patients should queue with the chronically ill is related to a positive attitude only in providing food assistance. TB-stigmatizing ideas are related to a positive attitude only in having special queues for TB patients. With respect to providing disability grants, TB-stigmatizing ideas show a negative attitude and those with the view that TB patients should queue with the chronically ill have a positive attitude.

There is unanimity of opinion on only one item: negative attitudes toward providing information at the patient's workplace or school were associated with both TB-stigmatizing ideas and the view that TB patients should queue with the chronically ill except for the higher educated. However, this agreement could be based on different reasons. People with TB-stigmatizing views may think that this type of assistance does not help, because TB patients have to help themselves and people with the view that TB patients should queue with the chronically ill might feel that TB patients do not wish to be recognized as TB patient. The view that TB patients benefit from being included with the chronically ill is related to positive attitudes toward supporting financial assistance. Providing porridge is a less obvious form of financial assistance than disability grants, but it alleviates a burden for both the TB patient and the family. Food is an immediate benefit and saves the family money. People with the combined-queuing view preferred financial assistance through porridge and grants rather than the more controlled assistance types such as DOTS volunteers, whose home visits may identify an individual as a TB patient. This may also be the main reason for preferring that TB patients be in the same line as the chronically ill. Chronic diseases are usually not associated with stigma; the visibility of seeking treatment for a chronic illness therefore was not associated with negative attitudes among respondents with this view.

The TB stigmatizing view also is related to contrasting attitudes toward TB treatment assistance. While those who favor combined queues for TB and other chronically ill patients found financial assistance types helpful, those with TB stigmatizing ideas had negative attitudes toward disability grants. People with stigmatizing ideas might blame TB patients for their illness and therefore believe they do not deserve financial support. A positive attitude was found between TB-stigmatizing ideas and the controlled observation of treatment by a DOTS volunteer. This view is related to preferences for visible-stigma-sensitive assistance types and negative attitudes toward financial assistance. Visibility of TB patients and exposure to stigma were not important factors for people with TB-stigmatizing ideas. They preferred separate queues for TB patients and direct observation of treatment by a DOTS volunteer. Results from a recent evaluation on effectiveness of TB treatment in four other TB crisis areas in South Africa showed that the fewer the patients allocated to DOTS volunteers, the higher the cure rate [21]. The same might apply for these communities. Visits from a DOTS volunteer lead to visible exposure within the neighborhood. This could explain the lower cure rates of the DOTS volunteer and suggest that complex behaviors must be taken into account when designing health policy.

### Study limitations

Given the cross-sectional nature of the results, interpretation of study results is restricted. Future research with a longitudinal approach would be valuable. Our analyses identified significant relations, but their relative strengths were often weak. We found a relationship between visibility factors and attitudes and preferences toward TB treatment but more research is necessary to include a larger set of visibility items. A major limitation is that our research investigated perceptions of the lay public only; behavior when faced with TB in a family member or friend might be different. However, given the random selection and inclusion of every tenth household, which led to equal probability of survey inclusion of lay perceptions in the community, and the fact that households with and without TB experience shared the same views indicate that our study findings might represent the actual situation. Since attitudes of the lay public play a central role in the patient's decision-making process it would be interesting to conduct a follow-up study to gain insight into whether and how disease stigmas actually influence an individual's attitudes and preferences toward TB treatment and assistance.

## Conclusions

Our results showed that two views related to visibility factors that expose patients to stigmatization (one characterized by TB stigma, the other by the view that TB patients should queue with other chronically ill patients) are associated with opposite attitudes and preferences towards TB treatment. Visibility factors related to the organization and delivery of treatment are also likely to affect health-seeking behavior; some patients might seek treatment at an earlier stage were it offered discreetly. Solutions are neither easy nor clear-cut, especially when considering highly-stigmatized diseases associated with complex behaviors. Various strategies are probably necessary to improve access and adherence to treatment and thereby health outcomes.

## Competing interests

The authors declare that they have no competing interests.

## Authors' contributions

JC and AN drafted the manuscript and performed the statistical analysis. Both authors have read and approved the final version of the manuscript.

## Appendix 1

### AIDS-related Stigma Scale

Please answer whether you agree or disagree with the following statements:

People who have AIDS are dirty

People who have AIDS are cursed

People who have AIDS should be ashamed

People with AIDS must expect some restrictions on their freedom

A person with AIDS must have done something wrong and deserves to be punished

People who have AIDS should be isolated

I do not want to be friends with someone who has AIDS

People who have AIDS should not be allowed to work

### TB-related Stigma Scale

Please answer whether you agree or disagree with the following statements:

People who have TB are dirty

People who have TB are cursed

People who have TB should be ashamed

People with TB must expect some restrictions on their freedom

A person with TB must have done something wrong and deserves to be punished

People who have TB should be isolated

I do not want to be friends with someone who has TB

People who have TB should not be allowed to work
